# Calcium-Activated Chloride Channels in Myometrial and Vascular Smooth Muscle

**DOI:** 10.3389/fphys.2021.751008

**Published:** 2021-10-15

**Authors:** Susan Wray, Clodagh Prendergast, Sarah Arrowsmith

**Affiliations:** ^1^Department of Women and Children’s Health, Institute of Life Course and Medical Sciences, University of Liverpool, Liverpool, United Kingdom; ^2^Department of Life Sciences, Manchester Metropolitan University, Manchester, United Kingdom

**Keywords:** calcium-activated chloride channels, vascular smooth muscle, myometrial smooth muscle, TMEM16A, Anoctamin 1, excitability, calcium, scramblases

## Abstract

In smooth muscle tissues, calcium-activated chloride channels (CaCC) provide the major anionic channel. Opening of these channels leads to chloride efflux and depolarization of the myocyte membrane. In this way, activation of the channels by a rise of intracellular [Ca^2+^], from a variety of sources, produces increased excitability and can initiate action potentials and contraction or increased tone. We now have a good mechanistic understanding of how the channels are activated and regulated, due to identification of TMEM16A (ANO1) as the molecular entity of the channel, but key questions remain. In reviewing these channels and comparing two distinct smooth muscles, myometrial and vascular, we expose the differences that occur in their activation mechanisms, properties, and control. We find that the myometrium only expresses “classical,” Ca^2+^-activated, and voltage sensitive channels, whereas both tonic and phasic blood vessels express classical, and non-classical, cGMP-regulated CaCC, which are voltage insensitive. This translates to more complex activation and regulation in vascular smooth muscles, irrespective of whether they are tonic or phasic. We therefore tentatively conclude that although these channels are expressed and functionally important in all smooth muscles, they are probably not part of the mechanisms governing phasic activity. Recent knockdown studies have produced unexpected functional results, e.g. no effects on labour and delivery, and tone increasing in some but decreasing in other vascular beds, strongly suggesting that there is still much to be explored concerning CaCC in smooth muscle.

## Introduction

Chloride (Cl^−^), its control, transport and contribution to fluid and volume control and excitability, is of long-standing interest to physiologists. It is abundant extracellularly in all cell types, at ~100–110mM, but of note, in smooth muscles, its concentration intracellularly is unusually high at 30–50mM, due to the activities of Cl^−^/HCO_3_^−^ exchanger and Na^+^K^+^Cl^−^ co-transporters. Consequently, when chloride channels open, chloride effluxes and the myocytes depolarize. In smooth muscles, there are volume-sensitive, bestrophins and CFTR chloride channels, but the most important, and the subject of this review, are Ca^2+^-activated chloride channels (CaCC). Unlike epithelial cells, in smooth muscle, CaCC are the major anion channel, but surprisingly much about CaCC remains enigmatic. Molecular and electrophysiological studies report CaCC properties that differ between tissues. By comparing two smooth muscles with different properties and regulatory mechanisms, myometrium and vascular, we will probe consistencies and differences in CaCCs. We will relate these findings to the functional roles and activation mechanisms of CaCCs. We start by summarising the background and current understanding of CaCCs, followed by an overview of the channels in smooth muscle, before moving on to the detailed review of them in myometrium and vascular smooth muscle (VSM).

## CaCC and the TMEM16 Family

Several reviews of CaCC composition, structure and regulation have been published and can be consulted for further details of the orientating overview we present here (Pedemonte and Galietta, 2014; Falzone et al., 2018; Kalienkova et al., 2021). The molecular identity of CaCC was established in 2008 (Caputo et al. 2008; Schroeder et al. 2008; Yang et al. 2008). TMEM16 were an orphan family of membrane proteins with 10 transmembrane domains (Brunner et al., 2014; Dang et al., 2017; Paulino et al., 2017a,b). This protein family was also called anoctamins, as they *were thought* to be anion-selective (An) and have eight (Oct) transmembrane domains. The topology and structure of CaCC are still being researched, but we know now, based on X-ray and cryo-EM data, that there are 10, not eight, transmembrane domains (Brunner et al., 2014; Paulino et al., 2017a). There are 10 members of the TMEM16 family and all are Ca^2+^-activated. TMEM16A and TMEM16B (ANO1 and 2) are the only pure anion channels, i.e. CaCCs, in the family (Suzuki et al., 2013). They form dimers (Fallah et al., 2011), with each monomer activated by Ca^2+^, thus forming a double-barrelled channel (Jeng et al., 2016; Lim et al., 2016; Paulino et al., 2017a,b). Most, if not all these other members of the TMEM16 family function predominantly as phospholipid scramblases, which are important for maintaining bilayer symmetry and function. Although still controversial, it appears that the scramblases can also be non-selective ion channels conducting cations, and in some cases anions (Yang et al., 2012; Grubb et al., 2013; Shimizu et al., 2013; Kim et al., 2018; Lin et al., 2018). Interestingly, with regard to this, there has been suggestions about whether CaCCs permit a degree (~15–20%) of cation flux [see discussions in Picollo et al. (2015); Falzone et al. (2018)]. Liposome studies of the pure CaCCs, however, show no dissipation of a KCl gradient supporting anion selectivity, as does the positive shift in reversal potential when SCN^−^ is substituted for Cl^−^ in the extracellular medium (Ferrera et al., 2011; Paulino et al., 2017a). It has been shown that single point mutations of the pore will greatly decrease anion selectivity and increase cationic (Yang et al., 2008, 2012; Peters et al., 2018). To explain this, it has been suggested that the CaCC channel has voltage-dependent conformational changes which may allow it to conduct cations (Peters et al., 2018). The details of the pharmacological and biophysical properties of native CaCCs vary depending on the tissues under study, possibly due to splice variations, heterodimers or even association of different subunits. Splicing leads to multiple isoforms and can have significant effects on channel function, such as changing Ca^2+^ sensitivity (Ferrera et al., 2009). Recently allosteric modulation of splice variants of CaCC by phosphatidylinositol 4,5-bisphosphate (PIP_2_) and CaMKII has been reported, adding to the complexity of channel regulation (Ko et al., 2020).

## Calcium-Activated Chloride Channels are Present in Smooth Muscles

Before the molecular identification of CaCC (Caputo et al., 2008; Schroeder et al., 2008; Yang et al., 2008), the presence of a Ca^2+^-activated Cl^−^ current (I_ClCa_) had been identified in SMCs, and functional studies conducted. Electrophysiological studies demonstrated slow (relative to L-type Ca^2+^ currents), voltage-dependent (at submaximal [Ca^2+^]), Ca^2+^-activated current, with outward rectification and corresponding functional studies showed depolarization and contraction [see (Large and Wang, 1996) for an excellent review of this early literature and (Janssen and Sims, 1992; Akbarali and Giles, 1993; Greenwood et al., 1995)]. A variety of not-so-specific CaCC inhibitors, including niflumic acid (NFA), 9-AC (9-Anthracenecarboxylic acid) and DIDS, (4,4’-Diisothiocyano-2,2ʹ-stilbenedisulfonic acid), blocked the current, produced hyperpolarisation and relaxed smooth muscles (Large and Wang, 1996). L-type Ca^2+^ channels (LTCC) represent the major pathway for the increase in Ca^2+^ needed for contraction in smooth muscles, but CaCCs can provide a positive feedback mechanism in the myocytes, as they are activated by the Ca^2+^ entry, and help to maintain depolarization as Cl^−^ effluxes through their pore.

The channels and currents have been found in all smooth muscle tissues, indicating an important functional role [Urethra (Drumm et al., 2021); bladder (Kajioka et al., 2004; Bijos et al., 2014); ureter (Iqbal et al., 2012; Hunziker et al., 2020); airway (Kotlikoff and Wang, 1998; Gallos et al., 2013); GI tract (Sanders et al., 2012); and oesophagus (Saha et al., 1992; Akbarali and Giles, 1993; Zhang and Paterson, 2002)]. There are, however, some key areas of uncertainty. With particular relevance to smooth muscles, (1) is the source of activating Ca^2+^ coming from the extracellular entry of Ca^2+^ (L-type and possibly TRP channels) or intracellular stores, i.e. the sarcoplasmic reticulum (SR) or mitochondria?, (2) is channel activation directly by Ca^2+^ or indirectly *via* a Ca^2+^-activated intermediary, e.g. calmodulin-activated kinases, and (3) are expression and functional effects in some smooth muscle tissues due to interstitial cells of Cajal (ICC), not myocytes, expressing the CaCC and passing the depolarization *via* gap junctions to the myocytes?

By comparing two types of smooth muscle, one spontaneous and phasic, and one tonic, we asked whether the answers to these three questions differed by type, but also if there was support for the suggestion that the contribution of CaCC to excitability also differed.

## Calcium-Activated Chloride Channels in Myometrium

**Figure 1:**
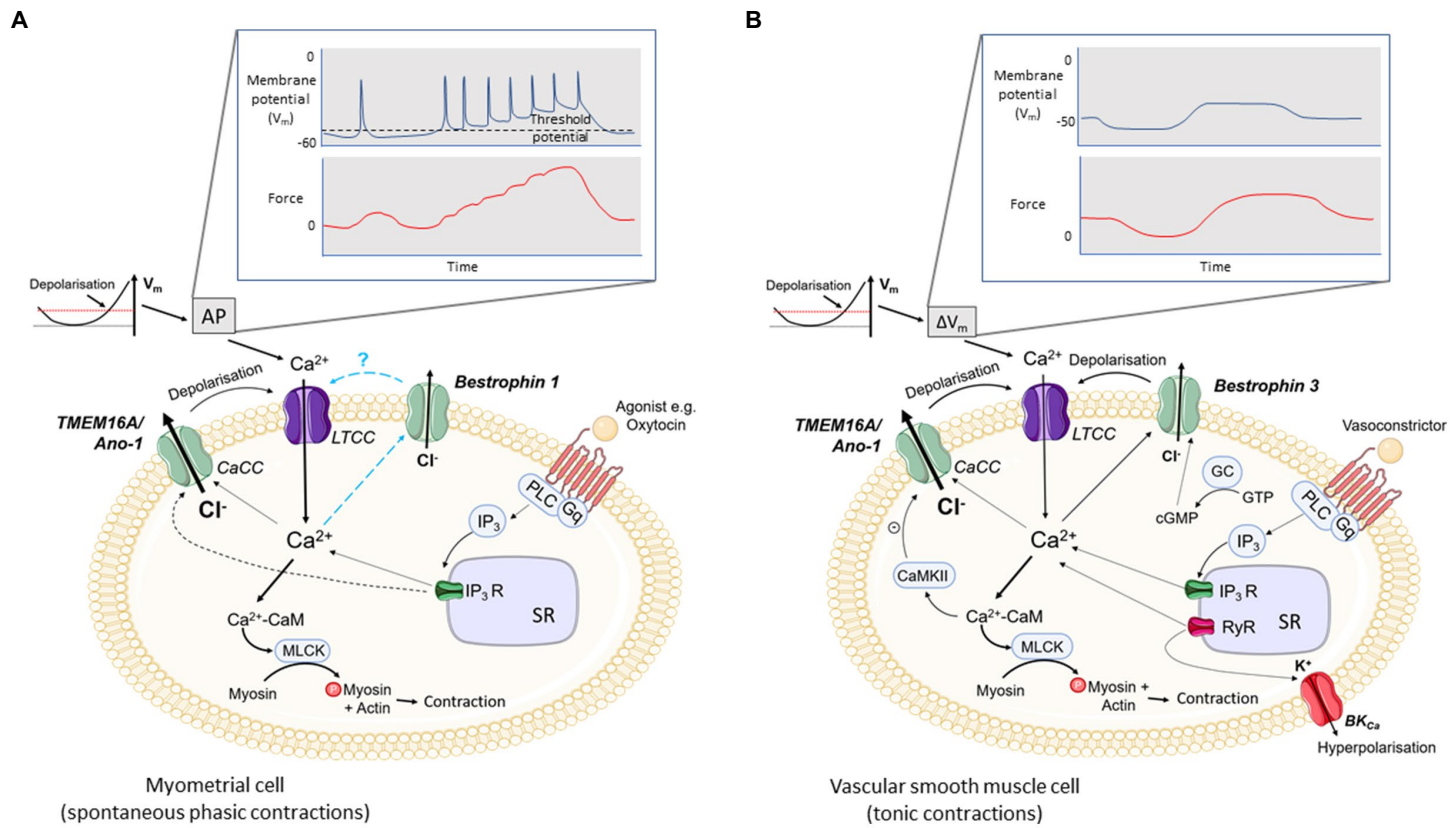
Excitation-contraction coupling in uterine and vascular smooth muscle cells (VSMCs) and the potential roles for CaCCs. **(A)** In spontaneously active smooth muscles, e.g. myometrium, depolarisation of the membrane potential (V_m_) to threshold initiates an action potential (AP) and phasic contractions. **(B)** In most vascular smooth muscles which are not spontaneously active, slow, tonic changes in V_m_ give rise to tonic contractions. In both, depolarisation leads to opening of voltage-gated calcium channels (L-Type, LTCC) and calcium entry. Calcium binds to calmodulin and the Ca^2+^-CaM complex activates myosin light chain kinase, MLCK. MLCK phosphorylates myosin promoting actin and myosin cross-bridge formation and contraction. Agonists, such as oxytocin in the myometrium **(A)** or vasoconstrictors in VSMCs **(B)**, binding to their receptor activate PLC which in turn hydrolyses PIP_2_ to IP_3_ and DAG (not shown). IP_3_ binds to its receptor, IP_3_R, on the SR and facilitates Ca^2+^ efflux into the cytoplasm. The rise in [Ca^2+^]_i_ is thought to activate calcium-activated chloride channels (CaCCs; TMEM16A/ANO-1 in myometrium and TMEM16A/ANO-1 and Bestrophin 3 in VSMCs), producing a chloride efflux, depolarisation of the cell membrane and opening of LTCCs. In myometrium, activation of CaCCs may be responsible for the initial depolarisation required to activate LTCCs. Ca^2+^ entry *via* LTCCs may also facilitate the opening of CaCCs which further increases the open probability of LTCC opening. The role of Bestrophins in excitation-contraction coupling in myometrium is not known. Additionally, in VSMCs, spontaneous calcium release through RyRs (Ca^2+^ sparks) on the SR can also contribute to CaCC opening (STICs) and depolarisation. Ca^2+^ sparks also activate BK_Ca_ leading to K^+^ efflux, STOCs and hyperpolarisation. Bestrophin channels are positively regulated by cGMP and TMEM16A/ANO1 channels are inhibited by CaMKII. Ca^2+^ entry *via* store – operated Ca^2+^ entry and *via* TRPC6 channels can also stimulate CaCC current and depolarisation (not shown).

The myometrium is a spontaneously active (myogenic) smooth muscle. The processes leading to cell activation and contraction are complex and involve the activity of several ion channels to promote a change in membrane potential (V_m_). The resting V_m_ of pregnant myocytes is around −60mV which shifts to −40mV towards term in rodents and humans (Parkington et al., 1999) in line with myometrial transition from quiescence during pregnancy to an actively contracting organ in labour. As is the case in other SMCs, membrane depolarization results in activation of L-type, voltage-gated Ca^2+^ channels (LTCCs) to provide calcium entry and action potential (AP) generation. The accompanying rise in [Ca^2+^]_i_ gives rise to contraction, and oscillations in V_m_ give rise to rhythmic contractions (Figure 1A).

The identity of the channel/s (and ions) responsible for the initial depolarisation to reach the threshold needed for LTCC activation is unknown. However, CaCCs have been implicated in carrying this current (Wray et al., 2015; Wray and Arrowsmith, 2021).

The presence of Cl^−^ currents in the myometrium was first described by Parkington and Coleman in single channel recordings in intact tissue strips of guinea-pig myometrium (Coleman and Parkington, 1987). A Cl^−^ current induced by oxytocin was also observed by Arnaudeau in rat myometrial cells following short-term culture (Arnaudeau et al., 1994). Later, our group, using freshly isolated rat myometrial cells, showed that this channel was activated by calcium entering *via* LTCC (Jones et al., 2004). The current was present in one-third of cells as a slowly deactivating tail current which was observed upon repolarisation following stepwise depolarisation. A tail current is the current remaining after the initial depolarizing stimulus has been removed and is an indicator of the timing of channel closures. That a tail current was observed following repolarisation when the LTCC are closed and L-type Ca^2+^ current would be inactive, suggested other channel species were present, open and carrying this late inward current. The current’s reversal potential was found to be close to that of Cl^−^ and was sensitive to [Cl^−^]. Moreover, it was sensitive to Ca^2+^ but not Ba^2+^ and was enhanced by the LTCC agonist, BayK8644 (Jones et al., 2004).

In addition to Ca^2+^ entry, other sources of activating Ca^2+^ could include that from the SR, either *via* local Ca^2+^ sparks from the spontaneous opening of Ryanodine receptors (RyR) or from a more global rise in Ca^2+^ *via* agonist-mediated IP_3_ release. However, calcium sparks do not occur in myometrium (Burdyga et al., 2007) and the RyRs are non-functional (Dabertrand et al., 2007). IP_3_ receptors are present and oxytocin is associated with activation of CaCCs (Arnaudeau et al., 1994). In cultured mouse myometrial cells, an inward current was recorded, which was blocked by CaCC inhibitors (Bernstein et al., 2014). The inhibitors also reduced agonist-mediated increases in Ca^2+^ and the authors suggested that SR release can also assist channel opening in myometrium (Bernstein et al., 2014). But whether SR Ca^2+^ release is a requirement for channel activation was not determined. As Ca^2+^ entry in the absence of an agonist was shown to activate CaCCs, Jones et al. concluded that L-type Ca^2+^ entry was the source of activating Ca^2+^. A similar logic would also hold for other intracellular organelles, such as mitochondria, but this has not been directly investigated.

Whether functional CaCC or other TMEM16 family members are present intracellularly, e.g. on SR or mitochondria is a controversial area but such expression has been reported in human myometrium (Pedemonte and Galietta, 2014; Danielsson et al., 2018). Whether there is a functionally important role for these channels within the SR or if their presence is simply due to protein processing and trafficking is not known.

Following the identification of TMEM16A and B (ANO1/2) and the development of TMEM16 A and B-selective inhibitors, their function as the putative CaCCs in myometrium has been studied. Both are expressed in human and rodent myometrium (Bernstein et al., 2014; Danielsson et al., 2015). Pharmacological inhibition of TMEM16A and B eliminated inward currents in patched mouse myometrial cells (Bernstein et al., 2014), produced hyperpolarisation in immortalised human myometrial cells [measured indirectly using a fluorescent potentiometric indicator and induced relaxation in human myometrial strips precontracted with oxytocin (Danielsson et al., 2018; Hyuga et al., 2018)]. siRNA knockdown of TMEM16A in primary or human myometrial cells also resulted in an attenuation of the oxytocin-induced increase in filamentous to globular actin ratio which is a marker of actin polymerisation and indicator of cell contraction (Danielsson et al., 2018). The findings from this group, albeit somewhat indirect and not on fresh myocytes, add to the suggestions of TMEM16A contributing to myometrial excitability.

### CaCC Function in Myometrium

That CaCCs are both voltage-gated and activated by increases in [Ca^2+^]_i_, makes them suitable candidates for participating in excitation-contraction coupling and AP generation in myometrium. The activation of CaCC will depolarize or maintain depolarization of the myometrial membrane, increase excitability and the open probability of LTCC, and AP generation. In this way, CaCCs in the myometrium are likely to contribute to both spontaneous and oxytocin-stimulated contractions with the latter thought to also involve IP_3_-mediated release of Ca^2+^ from the SR (Figure 1A).

The frequency, amplitude and duration of contractions have long been associated with the frequency and duration of AP firing (Burdyga et al., 2007, 2009). In rat myometrial tissues, CaCCs have also been implicated in stabilising the plateau phase of the AP and being responsible for prolonging the duration of contraction (Young and Bemis, 2009). A role for CaCCs in establishing or contributing to cell excitability is further supported by the reduced frequency of contraction following the application of several CaCC inhibitors (Yarar et al., 2001; Kaya et al., 2002; Jones et al., 2004; Bernstein et al., 2014; Danielsson et al., 2018; Hyuga et al., 2018). As mentioned previously, some CaCC blockers are non-specific, and off-target actions may synergise with their inhibition of CaCC to produce myometrial relaxation (tocolysis).

Interestingly, I_ClCa_ was only present in around one-third of freshly isolated rat myometrial cells (Jones et al., 2004), and only 5% of cultured murine cells showed auto-rhythmicity (Bernstein et al., 2014). This suggests that those cells expressing CaCC could be the pacemakers or electrogenic cells in the myometrium, equivalent to the ICC cells in gastric smooth muscle. Modelling of excitation-contraction coupling events by integrating transcriptomic data with electrophysiological data also supports a role for CaCCs in generating spontaneous depolarisations (Atia et al., 2016). Tantalising as these data are, direct evidence that CaCC are a major contributor to pacemaking and spontaneous activity in myometrium is lacking. Furthermore, TMEM16A expression was found to be downregulated (15-fold) in late gestation (non-labouring) pregnant human myometrium compared to non-pregnant (Danielsson et al., 2018). This reduced expression may reflect that the uterus is in a state of quiescence required to maintain pregnancy. Alternatively, TMEM16A channels may not be as important as suggested; SMC-specific deletion of TMEM16A in pregnant mice did not alter calcium signalling, uterine contraction or change the length of gestation (Qu et al., 2019), which casts doubt on the importance of the channel in myometrial physiology and its role in pacemaking for spontaneous, or as a depolarising channel for agonist-induced contractions. However, the extent of the reduction in TMEM16A in these conditional knockout mice is not clear.

Other channels known to display Ca^2+^ sensitivity and conduct Cl^−^ include Bestrophins (BEST 1, 2 and 3). Unlike TMEM16A/ANO1, however, they are not voltage sensitive. In expression systems, BEST-1 has been shown to modulate the LTCC current (Reichhart et al., 2010) by interacting with the channel’s β-subunits and regulating the number of pore forming subunits (Milenkovic et al., 2011). Its combined role as a Ca^2+^-dependent anion channel and regulator of LTCCs is thought to provide a feedback mechanism to control Ca^2+^-dependent Cl^−^ transport (Milenkovic et al., 2011). BEST-3 is a CaCC, which displays cGMP dependence [see later, Matchkov et al. (2008)]. BEST-1 is expressed in non-pregnant rat myometrium (Mijuskovic et al., 2015) and inhibition of bestrophins using DIDS has been implicated in mediating the relaxatory effect of hydrogen sulphide in myometrium (Mijuskovic et al., 2015). Their role in excitation-contraction coupling in myometrium, however, has not been studied.

Questions that remain unanswered include whether there is upregulation of TMEM16A or B in labour in humans to facilitate a pro-contractile drive, or similarly, whether expression and/or function of the channel is altered in preterm labour, and whether its inhibitors relax the labouring uterus. This is particularly important if they are to be used as targets for tocolysis – a major goal of the research in this area. Of note, both intra and extracellular protons have been shown to regulate TMEM16A activity (Cruz-Rangel et al., 2017): raising extracellular protons and subsequent channel protonation increase TMEM16A activation without changing their Ca^2+^ sensitivity. During contractions, the myometrium undergoes transient acidification (Larcombe-Mcdouall et al., 1999), which we have shown increases myometrial activity (Pierce et al., 2003; Alotaibi et al., 2015). Could CaCCs therefore, contribute to the stimulation seen under extracellular acidic conditions in labour?

## Summary

In myometrium, TMEM16A CaCC are expressed. We conclude that (1) L-type Ca^2+^ entry is the major source of activating calcium, (2) that this activation is direct, and (3) their expression is on myocytes, but only a subset of those present. More direct studies on labouring and non-labouring myometrium are urgently needed.

## Calcium-Activated Chloride Currents in VSM

Most VSM exists in a state of partial contraction (myogenic tone) from which it can dilate or constrict according to physiological requirements. There are some exceptions, such as portal vein, which exhibit spontaneous phasic contractions. Here, we will concentrate primarily on tonically contracting vessels as a comparison to the spontaneously active myometrial SM. However, as important work has been carried out in the phasic portal vein (which is arterial to the liver), and data on myometrium are limited, where relevant we will discuss data on both types of VSM and compare to the myometrium.

Ca^2+^-activated Cl^−^ currents (I_ClCa_) were first reported in VSM cells isolated from guinea-pig pulmonary artery (Byrne and Large, 1987). Since then, I_ClCa_ has been identified in many different tonic vessels (e.g. aorta, coronary, pulmonary, cerebral and mesenteric arteries) from multiple species (Lamb et al., 1994; Large and Wang, 1996; Davis et al., 2010, 2013; Manoury et al., 2010; Thomas-Gatewood et al., 2011; Matchkov et al., 2013; Dam et al., 2014; Cil et al., 2021). The currents have also been shown in spontaneously active vessels, e.g. in rabbit portal vein (Byrne and Large, 1988) and guinea-pig mesenteric vein (Van Helden, 1991).

The tail current amplitude varies between vessels and species: I_ClCa_ is large in conduit vessels (tail current density 40pA/pF) and very large in the pericytes of the microvasculature (130pA/pF), but small (5–10pA/pF) or absent in small arteries of the mouse (Heinze et al., 2014). However, other studies have identified these currents in rat, rabbit and human small arteries [5–10pA/pF in rabbit pulmonary and coronary arteries (Greenwood et al., 2001) and 20pA/pF in rat cerebral arteries (Thomas-Gatewood et al., 2011)], although current density was not shown in all (Klockner and Isenberg, 1991; Thomas-Gatewood et al., 2011; Dam et al., 2014). In myometrium, the amplitude of the tail current was 162±48pA in rat myocytes, equivalent to a current density of approx. 1.5pA/pF (Jones et al., 2004), which is small compared to the current in the SM of conduit vessels and microvessels. A similarly small peak current density of 5pA/pF was recorded in murine portal vein (Ohshiro et al., 2014). In spontaneously active venous tissues, spontaneous depolarizations are observed that have been linked to I_ClCa_ (Van Helden, 1991). ICC-like cells have been identified in portal vein (Povstyan et al., 2003; Huang et al., 2010), but it is not clear whether they act as pacemaker cells, as SMC also generate spontaneous depolarizations. Interestingly, it was found that 40% of portal vein myocytes express an I_ClCa_ compared to 90% of myocytes from the tonic pulmonary and coronary arteries (Greenwood et al., 2001), an expression difference perhaps linked to the differential nature of a tonic vessel and spontaneously active vessel with pacemaker activity.

### CaCC Identity in VSM

TMEM16A/ANO1 has been identified as the ‘classical’ CaCC in VSM and TMEM16A mRNA and protein are widely expressed (Davis et al., 2010; Manoury et al., 2010; Thomas-Gatewood et al., 2011). Inhibition of CaCC by traditional non-selective blockers [e.g. NFA (Criddle et al., 1996, 1997; Kamouchi et al., 1997; Sun et al., 2012)] or more recent selective inhibitors [e.g. T16A_inh_-A01 and TM_inh_-23 (Davis et al., 2010, 2013; Cil et al., 2021)] leads to vasorelaxation of tonic VSM. siRNA knockdown of TMEM16A also reduces arterial constriction (Bulley et al., 2012; Dam et al., 2014; Heinze et al., 2014; Jensen et al., 2018).

Multiple splice variants of TMEM16A have been identified in murine thoracic aorta, carotid artery and portal vein (Davis et al., 2010; Ohshiro et al., 2014). Splice variants can exhibit differing Ca^2+^ sensitivities and voltage-dependence (Ferrera et al., 2009). In portal vein, Ohshiro and co-workers (Ohshiro et al., 2014) have demonstrated the ability of splice variants to homo and hetero-dimerize. Varied co-expression of splice variants and homo/heterodimerization of channels provides great scope for diversity of physiological functioning of CaCC across different vascular beds.

Many VSMs also co-express a second Ca^2+^-dependent Cl^−^ conductance that is distinct from the classical I_ClCa_, in that it requires cGMP for activation, is not voltage-dependent and is resistant to inhibition by NFA (Matchkov et al., 2005). Both channels co-exist, but relative distribution varies along the vascular tree (Matchkov et al., 2005). This I_Cl(cGMP,Ca)_ current is encoded by bestrophin 3, rather than the TMEM16A/ANO1 gene, since knockdown of the bestrophin gene leads to disappearance of the cGMP-dependent chloride current, but not the classical I_ClCa_ current (Matchkov et al., 2008; Broegger et al., 2011). I_Cl(cGMP,Ca)_ does not mediate agonist-stimulated contraction but instead plays a role in regulation of tissue perfusion by mediating tone oscillations in VSM (Boedtkjer et al., 2008; Broegger et al., 2011). Both cGMP-dependent and classical I_ClCa_ currents are expressed in spontaneously active portal vein (Matchkov et al., 2005). This differs from myometrium, where only classical CaCC are expressed and BEST-3 has not been reported, but BEST-1 has (Mijuskovic et al., 2015). Thus, I_ClCaGMP_ is a feature of VSM – irrespective of it being phasic or tonic.

### CaCC Function in VSM

Similar to myometrium and other SM, CaCC play a role in excitation-contraction coupling in VSM cells (Large and Wang, 1996). The Ca^2+^ required to activate CaCC in tonic VSM can come from multiple sources including extracellular and agonist- (e.g. vasoconstrictor) mediated SR release (Figure 1B). Compared to myometrium, in VSM, the mechanisms that have been shown to activate CaCC are more varied. Depolarising currents that activate Ca^2+^ entry *via* LTCC trigger I_ClCa_, either directly [coronary artery (Lamb et al., 1994), renal artery (Gordienko et al., 1994)] or by stimulating Ca^2+^-induced Ca^2+^ release *via* RyR (Lamb et al., 1994). Agonist-mediated release of Ca^2+^ from the SR [pulmonary artery (Helliwell et al., 1994; Yuan, 1997)] is another important route by which I_ClCa_ is activated. Ca^2+^ entry *via* store-operated Ca^2+^ entry (SOCE) [pulmonary artery (Forrest et al., 2010; Angermann et al., 2012)] and *via* TRPC6 channels [cerebral artery (Wang et al., 2016)] can also stimulate CaCC current and depolarisation.

In VSM, the spontaneous release of Ca^2+^ from the SR *via* RyRs (Ca^2+^ sparks) is associated with stimulation of Ca^2+^-activated channels on the sarcolemma. When large-conductance Ca^2+^-dependent K^+^ channels (BK_Ca_) are activated in this way, spontaneous transient outward currents (STOCs) are generated, and hyperpolarisation and vasodilation ensue (Nelson et al., 1995). When CaCC are activated, spontaneous transient inward currents (STICs) occur and cause depolarisation and vasoconstriction, thus playing a role in determining vasomotor tone. The balance between these two pathways varies from vessel to vessel and will determine the overall contractility of the VSM [cerebral artery (Zhao et al., 2017) and renal arterioles (Yip et al., 2018)]. Ca^2+^ sparks and associated CaCC-mediated STICs have been identified in both tonic and phasically contracting VSMs (Wang et al., 1992; Large and Wang, 1996; Yip et al., 2018).

Diverse sources of Ca^2+^ activate I_ClCa_ in the spontaneously active portal vein preparation [L-type Ca^2+^ entry, agonist-induced SR Ca^2+^ release and spontaneous release of Ca^2+^ from the SR (sparks) and reverse mode sodium-calcium exchange (NCX)] (Wang et al., 1992; Leblanc and Leung, 1995; Greenwood and Large, 1996; Burt, 2003; Saleh and Greenwood, 2005). In contrast, few mechanisms of I_ClCa_ activation have been identified in the spontaneously active myometrial smooth muscle (Figure 1A). As mentioned above, in the myometrium, Ca^2+^ sparks are not found and RyRs are non-functional. Nor have the role of NCX, SOCE, TPRC6 been determined in relation to CaCC activation.

In addition to mediating the response to contractile agonists, CaCC are involved the vascular myogenic response. CaCC antagonists or TMEM16A knockdown hyperpolarises and dilates vessels (Nelson et al., 1997; Bulley et al., 2012; Yip et al., 2018) and reducing extracellular Cl^−^ augments myogenic tone (Doughty and Langton, 2001). CaCC are also implicated in the generation of vasomotion (Boedtkjer et al., 2008; Dam et al., 2014) and spontaneous contraction of portal vein (Wang et al., 1992).

Vascular smooth muscle-specific disruption of TMEM16A/ANO1 in mice abolished I_ClCa_ in VSM of the aorta, carotid artery and small arterioles of the brain and retina and resulted in lowering of blood pressure (Heinze et al., 2014). This hypotensive effect is likely mediated *via* small diameter arterioles, where many CaCC are expressed and the vasocontractility they mediate will affect peripheral resistance. In the spontaneously hypertensive rat, TMEM16A/ANO1 is overexpressed in aorta, carotid, mesenteric and hind limb arteries (Wang et al., 2015). siRNA knockdown or pharmacological inhibition of these channels prevented hypertension development in this model (Wang et al., 2015). In addition, TMEM16A expression and activity are significantly upregulated in various pulmonary hypertension models (Forrest et al., 2012; Sun et al., 2012; Papp et al., 2019). However, in an alternative model of hypertension, the 2 kidney 2 clip model, TMEM16A expression and I_ClCa_ are reduced and CaCC activity is negatively correlated with blood pressure and medial cross-sectional area of the basilar artery (Wang et al., 2012), suggesting that downregulation of CaCC is associated with the cerebrovascular remodelling that occurs during hypertension.

In a recent study of heterozygous TMEM16A/ANO1 knockout mice, a 50% decrease in I_ClCa_ reduced aortic contractility as expected, but paradoxical increases in tail/saphenous artery contractility were observed (Matchkov et al., 2020). Clearly the *in vivo* functional role of CaCC is complicated and requires further examination.

### Regulation of Classical CaCC in VSM

TMEM16A/ANO1 channels are gated by Ca^2+^, but sustained Ca^2+^-activation induces desensitisation. When TMEM16A is expressed in HEK293T cells, PIP_2_ is required for channel activation and guards against Ca^2+^-induced inactivation (Arreola and Hartzell, 2019; Le et al., 2019). This means that agonist-induced IP_3_ production on the one hand activates CaCC, but simultaneously PIP_2_ hydrolysis reduces the availability of PIP_2_ and reduces channel opening. Similar findings were reported in detrusor SMCs (Yarotskyy et al., 2019); however, the only study to examine the role of PIP_2_ in VSM found that PIP_2_ tonically inhibits CaCC (Pritchard et al., 2014). Further investigations are required to determine whether PIP_2_ is inhibitory in all VSM and why the mechanism differs between cell types.

Phosphorylation by Ca^2+^-calmodulin-dependent kinase II (CaMKII) attenuates activation of CaCC in a variety of VSMs (Greenwood et al., 2001; Wiwchar et al., 2009). This represents another important negative feedback mechanism whereby vasoconstricting agonists can limit the depolarising influence of CaCC. Calmodulin mediates the Ca^2+^-dependent inactivation of TMEM16A (Yang et al., 2014; Yang and Colecraft, 2016) but not its activation. Since phosphorylation inactivates CaCC, phosphatases, such as calcineurin, PP1 and PP2, can enhance channel function (Ledoux et al., 2003; Ayon et al., 2009).

Although TMEM16A expression is relatively evenly spread across the VSM sarcolemma (Davis et al., 2010), it is suggested that TMEM16A are to some extent localised to lipid microdomains, as the amplitude and pharmacology of I_ClCa_ are significantly altered by cholesterol depletion with methyl-β-cyclodextrin (Sones et al., 2010), although this has only been studied in mouse portal vein thus far. There is an intriguing overlap in pharmacology between BK_Ca_ channels and CaCC (Greenwood and Leblanc, 2007), which may be partly related to their co-localisation in lipid rafts given a loss of shared pharmacology after cholesterol depletion (Sones et al., 2010), although it is likely more complicated than simple co-localisation.

As mentioned earlier, changes in pH affect channel activity (Cruz-Rangel et al., 2017) and in smooth muscles, this may lead to changes in cell excitability. In pulmonary artery myocytes, chronic hypoxia, which will also change pH, strongly increases TMEM16A expression and CaCC currents, which may be the cause of the enhanced serotonin-mediated vasoreactivity associated with pulmonary hypertension (Sun et al., 2012).

### Comparison of Tonic and Phasic Smooth Muscles

We have described CaCC expression, function and regulation in two very different smooth muscles: the myometrium and tonic VSMs, with some comparisons made to spontaneously active VSM. Returning to the questions we posed at this start of this review, in relation to the source of CaCC-activating Ca^2+^, there appears to be genuine, rather than experimental differences between smooth muscles, e.g. in uterus, L-type Ca^2+^ entry is the activator, whereas in blood vessels, SR Ca^2+^ release through RyRs (and other alternative sources, e.g. SOCE) is also important. In myometrium, any SR Ca^2+^ activating the CaCCs will be from agonist-mediated release through IP_3_Rs. Other smooth muscles not discussed in this review also show these differences [e.g. urethra (Drumm et al., 2018)].

Whether channel activation is directly by Ca^2+^ or *via* an intermediary, e.g. CaM kinase II, has been a controversial area. Evidence to support direct binding of Ca^2+^ activating the channel has come from expression of human TMEM16A in liposomes (Terashima et al., 2013), in which all the properties of CaCC, including Ca^2+^ sensitivity, were recapitulated in the absence of calmodulin. The review Yang and Colecraft (2016) concluded that ‘there is now overwhelming evidence that Ca^2+^-dependent activation occurs through CaCC binding directly to the channel, and does not require CaM’. In fact, phosphorylation by CaMKII attenuates activation of CaCC in many VSMs (Greenwood et al., 2001; Wiwchar et al., 2009) and to date, such regulation has not been studied in myometrium.

In phasic smooth muscles, a major question is around the identity of the cells expressing and conducting the CaCC current. In the GI tract, CaCC channel expression and pacemaking activity are exclusively in the ICC cells and in urethra, both myocytes and ICCs express the channel (Sanders, 2019). ICC-like cells have been identified in human uterus (Duquette et al., 2005), but their role in uterine contractile activity is still unclear. A third of rat myometrial SM cells express an I_ClCa_ (Jones et al. Pusch, 2004): but are these then the myometrial pacemaker cells? As mentioned above, ICC-like cells have also been identified in portal vein (Povstyan et al., 2003; Huang et al., 2010), but since both the ICC and SMC generate spontaneous rhythmic inward currents, it is not clear exactly where the pacemaker activity lies. It is interesting however that I_ClCa_ is confined to 40% of portal vein myocytes (Greenwood et al., 2001), a similar number to myometrium, whereas the majority of cells in tonic vessels expresses I_ClCa_.

Most VSM express both the classical I_ClCa_ (mediated by TMEM16A) and a cGMP-dependent I_Cl(cGMP,Ca)_ (mediated by BEST-3). So far myometrium has also been shown to express the classical I_ClCa_ (TMEM16A) and although BEST-1 is expressed, BEST-3 and I_Cl(cGMP,Ca)_ have not yet been identified.

Although the data are far from exhaustive or always in agreement, we consider that CaCCs cannot explain the differences in electrical activity and hence contraction, between different smooth muscles.

## Conclusion and Future Directions

Calcium-activated chloride channels are an important aspect of SMC physiology, particularly their contribution to excitation and regulation of contraction. Targeting them pharmacologically to modulate their activity is an attractive goal and may aid treatment of several SMC disorders including preterm labour and hypertension. The identification of TMEM16A as the channel-forming protein has enabled deeper insight into their role and expression in several SMCs and in different species, which has brought us somewhat closer to achieving this aim. However, as we have discussed, differences in their role (e.g. whether CaCCs participate in initial depolarisations and AP generation), their activation (e.g. from L-type entry as seen in uterus, or SR Ca^2+^ release, or both as seen in VSMs) and their regulation exist between different SMs and between species, so we cannot simply extrapolate or generalise findings. The non-specificity of classical CaCC inhibitors was problematic for many years, but the recent development of more selective inhibitors should aid the ongoing elucidation of the role of CaCC in smooth muscles. The role of TMEM16A splice variants and their effect on function needs further elucidating and appears not to have been examined in the myometrium. That TMEM16A mutations are associated with a range of pathologies is also interesting and may point towards future pharmacogenetic profiling or personalised medicine approaches.

The localisation of CaCCs to intracellular membranes including the SR is interesting given the repertoire of Ca^2+^ release channels present and begs more questions into their role there. Could SR TMEM16A interfere with the function of these Ca^2+^-release channels? Could they provide a counter current to SR Ca^2+^ release and/or determine the rate of cytosolic Ca^2+^ increase? Further work is also needed to determine their involvement and interaction with other signalling proteins and channels within membrane microdomains, as well as understanding more about how endogenous modulators or changes to the extracellular milieu can regulate their function.

## Author Contributions

SW, CP, and SA contributed equally to the writing of the manuscript and have approved the submitted version.

## Funding

This work was supported by the University of Liverpool and the Manchester Metropolitan University should be credited as funders.

## Conflict of Interest

The authors declare that the research was conducted in the absence of any commercial or financial relationships that could be construed as a potential conflict of interest.

## Publisher’s Note

All claims expressed in this article are solely those of the authors and do not necessarily represent those of their affiliated organizations, or those of the publisher, the editors and the reviewers. Any product that may be evaluated in this article, or claim that may be made by its manufacturer, is not guaranteed or endorsed by the publisher.

## Abbreviations

AP: action potential
SMC: smooth muscle cell
VSM: vascular smooth muscle
CaCC: Ca^2+^-activated chloride channel
PIP_2_: phosphatidylinositol 4,5-bisphosphate
LTCC: L-type Ca^2+^ channel
MLCK: myosin light chain kinase
PLC: phospholipase C
DAG: diacylglycerol
TMEM16A, ANO: anoctamin
SR: sarcoplasmic reticulum
STIC: spontaneous transient inward current
STOC: spontaneous transient outward current
CaMKII: Ca^2+^-calmodulin kinase II
